# 2D to 3D Magnetism in Synthetic Micas

**DOI:** 10.1002/advs.202408266

**Published:** 2024-09-20

**Authors:** José Luis Rosas‐Huerta, Jonas Wolber, Claire Minaud, Oscar Fabelo, Clemens Ritter, Olivier Mentré, Ángel M. Arévalo‐López

**Affiliations:** ^1^ Unité de Catalyse et Chimie du Solide (UCCS) – Université de Lille – Centrale Lille Université Artois ENSCL, UMR CNRS 8181 Lille F‐59000 France; ^2^ Institut Laue‐Langevin BP 156 Grenoble Cedex 38042 France

**Keywords:** layered materials, low‐dimensionality, magnetism, micas, properties

## Abstract

Fe‐based mica minerals usually display two opposing magnetic ground states, either they behave as spin‐glasses or as layered ferrimagnets. No definite reason has been proposed as an explanation for this duality. This conundrum is unraveled by comparing the synthetic micas KFe_3_[*M*Ge_3_]O_10_ *X*_2_ with *M*═Fe and Ga, *X*═OH^−^ and F^−^. Neutron diffraction demonstrates a 2D to 3D magnetic transition in KFe_3_[FeGe_3_]O_10_(OH)_2_ while just hints or no order at all are observed for the fluorides with *M*═Fe and Ga respectively. The 3D transition is triggered by the presence of iron in the intralayer tetrahedra. DFT+U calculations show that the magnetic exchange couplings between the previously believed solely magnetic octahedral layers would otherwise be frustrated without this intralayer iron.

## Graphical abstract

2D to 3D magnetic transition in KFe_3_[FeGe_3_]O_10_(OH)_2_ synthetic mica is observed. This occurs thanks to the intralayer tetrahedral Fe, which couples antiferromagnetically with the otherwise frustrated octahedra layer.

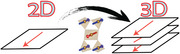

## 1 Introduction

Among low‐dimensional magnetic inorganic materials, those built on triangular iron oxide layers are prone to unusual properties. For instance, they can order ferromagnetically (FM) with in‐plane and out‐of‐plane magnetic easy‐axes as respectively observed in the Fe(OH)_2_^[^ ^1^ ^]^ and Fe(Cl)_2_^[^ ^2^ ^]^ van der Waals materials and thus displaying half‐metallic FM single‐layers.^[^ ^3^ ^]^ In addition, the orbital degeneracy offered by the Fe^2+^ coordination in some layered compounds is responsible for their giant magneto‐crystalline anisotropy, as in the 2D‐Ising FM BaFe^2+^_2_(PO_4_)_2_ and the multiferroic LuFe^2.5+^_2_O_4_. In these, the anisotropy induces the freezing of magnetic domains at low‐temperatures with record magnetic coercivities (H_c_ > 20 T).^[^ ^4^, ^5^, ^6^ ^]^ These recent results stimulate this work in the search of genuine iron oxides with robust uniaxial FM 2D‐subunits.

**Figure 1:**
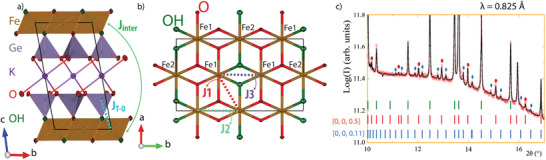
a) Crystal structure of KFe_3_[FeGe_3_]O_10_(OH)_2_. b) *ab* plane shows the triangular layer created by the Fe octahedra with OH *cis* and *trans* configurations for Fe1 and Fe2 respectively. Magnetic exchange interactions *J_i_ i =* 1, 2, 3, inter, and T‐O are labeled. c) Enlarged region of the SXRD Rietveld refinement at 300 K (λ = 0.825 Å) with red and blue marks indicating 2*c* and 9*c* supercells.

As the worldwide production of mica is 2.1 and 3.6 times larger with respect to Co and Li metals respectively, they are an important class of minerals with commercial use in aerospace, electronics, automotive, cosmetic industries, etc.^[^ ^7^ ^]^ With an *AB*_3_(*C*)_4_O_10_ *X*_2_ equation, their general structural description dates back to Pauling.^[^ ^8^ ^]^ It consists of a 2D triangular lattice of edge‐sharing octahedra (BO_4_ *X*_2_, *X*═OH^−^ or F^−^) in between two opposing tetrahedral layers (*C*). These 2:1 layers are separated by large cations such as *A*═K^+^, Na^+^, or Ca^2+^, see **Figure** **1**a. The occupancy of the octahedra defines two groups: i) so‐called trioctahedral series with a triangular lattice fully occupied by a divalent cation (*B*═Mg^2+^ or Fe^2+^) and ii) so‐called dioctahedral series with 2/3 of the octahedra occupied and defining a honeycomb lattice filled with a trivalent cation (*B*═Al^3+^ or Fe^3+^). The tetrahedra usually contains *C*═Si^4+^, Al^3+^, or Fe^3+^. The long distance in between octahedra layers (≈10 Å) along with the presence of Fe^2+^ in the trioctahedral micas reveal them as potential 2D‐magnetic materials.

Most of the magnetic properties studies of micas have been realized in mineral samples which involved complicated compositions with different degrees of doping in both octahedral and tetrahedral sheets.^[^ ^9^ ^]^ Nevertheless, two types of opposed magnetic behavior are usually observed: i) a spin‐glass transition^[^ ^10^, ^11^ ^]^ or ii) a 3D magnetically ordered state within octahedral sheets.^[^ ^12^, ^13^ ^]^

Herein, we have discovered the KFe_3_^2+^[FeGe_3_]O_10_(*X*)_2_ (*X*═OH^−^ and F^−^) trioctahedral synthetic mica with a 2D to 3D transition. We also demonstrate the crucial role of iron within the tetrahedral site to develop 3D ordering thus providing a new direction for the use of this important family as magnetic materials.

## 2 Results and Discussion

Powder and single crystalline KFe_3_[FeGe_3_]O_10_(OH)_2_ samples were synthesized in a high‐pressure autoclave at 773 and 1.7 kbar (experimental details are given in Supporting Information). The structure was solved through X‐ray diffraction analysis of a small single crystal (CSD‐2369861) with a pseudo‐trigonal symmetry. The refinement results are given in Table S1 (Supporting Information) and the structure is shown in Figure 1a. Synchrotron radiation confirmed the bulk structure. However, small reflections indexed as 2*c and 9*c supercells were observed in the bulk but not considered in the structural modeling, see Figure 1c, such reflections were not detected in the neutron data detailed later. These superstructures nucleate from different stacking sequences as observed in the trioctahedral mica‐related mineral biotite K(Mg, Fe)_3_Si_3_AlO_10_(OH, F)_2_.^[^ ^14^ ^]^

Bond Valence Sum (BVS) estimates of the oxidation states of the different sites, which shows that the tetrahedra contain Fe^3+^/Ge^4+^ while the octahedra layer has Fe^2+^ in a (FeO_4_(OH)_2_) coordination with a *cis*(Fe1) – *cis*(Fe1) – *trans*(Fe2) OH^−^ configuration sequence along *b*, see Figure 1b. The well‐known anionic ordering in micas is directly imposed by the upper and lower tetrahedral layers which define a (FeGe_3_O_10_) hexagonal ring which is solely formed by oxygen and leaves the place at the center for the hydroxyl/fluoride anion to coordinate with only 3 metals.

**Figure 2:**
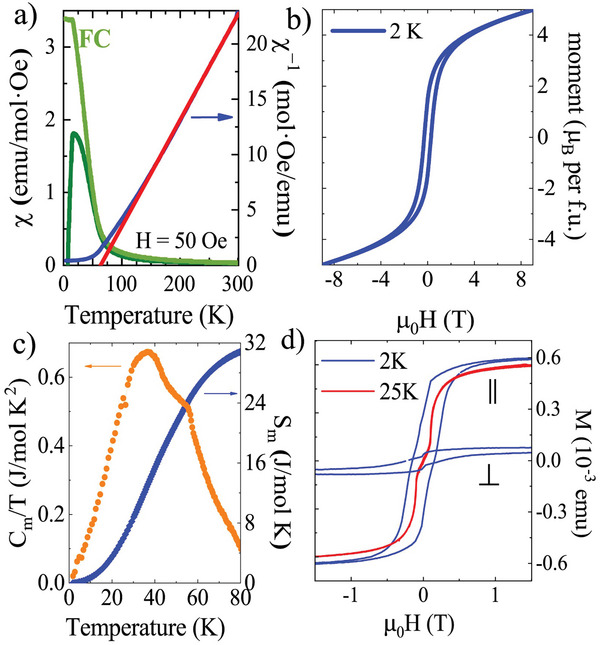
a) FC/ZFC magnetic susceptibilities for KFe_3_[FeGe_3_]O_10_(OH)_2_ and inverse ZFC data with Curie‐Weiss fit to 150–300 K points, giving the paramagnetic moment at high temperature, is shown with a red straight line. b) M vs H at 2 K in a powder sample. c) Thermal evolution of the magnetic contribution to the heat capacity divided by temperature (C_m_/T) and the calculated magnetic entropy (S_m_). d) M vs H with parallel (‖) and perpendicular (⊥) field to the *ab* plane at 2 K (blue) and 25 K (red) for one single crystal. Weight of the crystal was unknown and therefore the used units.

Low‐temperature magnetic susceptibility for KFe_3_^2+^[FeGe_3_]O_10_(OH)_2_ synthetic mica is shown in **Figure** **2**a. A Curie‐Weiss fit to 150 – 300 K temperature range gives an effective moment of 9.10(6) µ_B_ per formula unit, slightly reduced from the theoretical value of 10.34 µ_B_ from three *S* = 2 Fe^2+^ and one 5/2 Fe^3+^ ground‐state contributions. This may be due to covalency effects in the triangular lattice (also observed via DFT, discussed later). A positive Curie‐Weiss temperature (*θ* = 63.5(1) K) shows that ferromagnetic interactions are dominant. However, the low remnant magnetization at 2 K in a powder sample of *M_s_* = 5.0(1) µ_B_ rules out a simple ferromagnetic spin alignment, see Figure 2b. The linear increase above 4 T evidences the robust antiferromagnetism (AFM) between octahedra and tetrahedra layers as discovered by neutron diffraction discussed below.

Single crystal magnetic hysteresis loop at 2 K shows a 5.6 M_‖_/M_⊥_ ratio at 9 T demonstrating a strong anisotropy, see Figure S4 (Supporting Information). A metamagnetic transition also occurs below 25 K and above 0.1 T when the field is applied parallel to the *ab* plane, see Figure 2d. A similar behavior was reported in natural biotite K(Mg, Fe)_3_[AlSi_3_]O_10_(OH, F)_2_.^[^ ^15^ ^]^ Magnetic correlations already observed from deviations of the CW‐fit in Figure 2a originate an increase to the magnetic contribution of the heat capacity below 100 K with a maximum at *T_incom_* = 56 K and a broad second transition with a maximum at *T_C_* = 40 K, see Supporting Information. The magnetic entropy contribution of *S_m_* = 32(1) J mol^−1^ K^−1^ accounts for ≈60% of the expected 55 J mol^−1^ K^−1^ for three Fe^2+^ (*S* = 2) and one Fe^3+^ (*S* = 5/2). This may be related to the magnetic frustration in the octahedral layer as discussed later.

**Figure 3:**
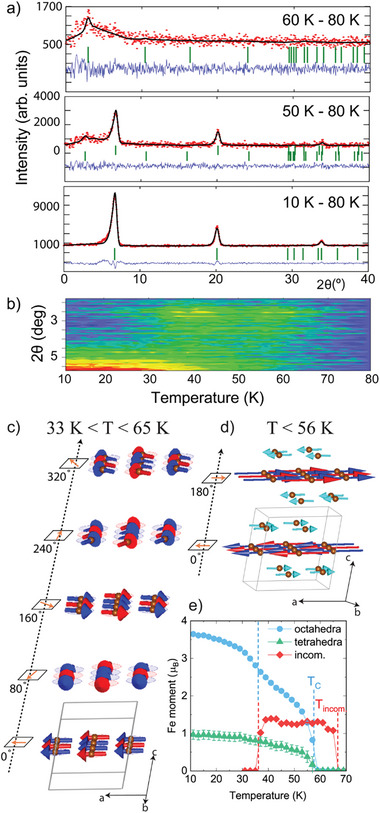
a) Rietveld refinements to NPD difference patterns at 60, 50, and 10 K minus 80 K. b) Thermal plot for the low angle NPD data. c) and d) Magnetic structures at 60 K and 1.5 K. e) Refined magnetic moments on the Fe sites. The tetrahedra moment is considered with its multiplicity, that is divided by 4.

Difference patterns of powder neutron diffraction data were used to explore the low‐temperature magnetic order in KFe_3_[FeGe_3_]O_10_(OH)_2_, see **Figure** **3**a. Between 65 and 35 K, a broad magnetic peak appears at ≈3° in 2θ, Figure 3b. The asymmetry of this maxima is highly reminiscent of a 2D system with short‐range correlations.^[^ ^16^, ^17^ ^]^ The correlation length of ξ ≈ 100 Å was obtained from a Warren function, see Supporting Information.^[^ ^17^ ^]^ However, the signatures in the heat capacity measurements point toward long‐range order (LRO) and the maxima was indexed with a *k_1_* = [0 0 0.22] incommensurate propagation vector. This incommensurate magnetic structure describes the octahedral Fe^2+^ layers ordered ferromagnetically but rotating ≈80° in between each other. A correlation length of ≈28 Å was obtained from the Scherrer formula which is ≈1/3 of the full incommensurate magnetic structure (≈90 Å along *c*), thus favoring the 2D description. Below 56 K, another set of maxima appears with *k_2_* = [0 0 ½]. Details of the refinement are given in Supporting Information. It describes the same planes stacked antiferromagnetically along *c*, that is with a 180° rotation in between layers. However, despite their 1/4^th^ disordered occupancy, the contribution of Fe^3+^ in the tetrahedra is also significant and the spins arrange antiferromagnetically to the octahedra layers, in agreement with Goodenough‐Kanamori‐Anderson rules.^[^ ^18^, ^19^, ^20^ ^]^ This behavior is similar to magnetite, Fe_3_O_4_, where the ferromagnetic sublattice relies on the 90° Fe^3+^‐O‐Fe^3+^ super‐exchanges assisted by Fe^3+^‐O‐Fe^2+^ double‐exchanges.^[^ ^21^ ^]^

The thermal evolutions of the refined moments for both structures are shown in Figure 3e. Below 35 K, the incommensurate structure disappears and a sudden increase of the magnetic moment in the octahedra for the *k_2_* structure occurs, confirming the transition from the incommensurate (2D – like) to the 3D LRO. The 1.5 K magnetic structure has moments of 3.72(1) µ_B_ and 4.03(3) µ_B_ for Fe^2+^ and Fe^3+^, close to the ideal values for *S* = 2 but slightly reduced for *S* = 5/2, possibly related to the disorder with germanium in the tetrahedra. This is consistent with the bulk magnetization since no saturation is reached at 2 K under 9 T, see Figure 2b.

To investigate the ansatz for the development from a 2D to a 3D magnetic structure complementary KFe_3_[*M*Ge_3_]O_10_F_2_ (*M*═Fe and Ga) micas were prepared and measured, see Supporting Information. Closely to the previously described KFe_3_[*M*Ge_3_]O_10_(OH)_2_, the oxyfluoride *M*═Fe sample gives hints of LRO with *k* = [0 0 ½]. However, the *M*═Ga oxyfluoride mica did not show low‐temperature magnetic diffraction maxima, independently of displaying similar magnetic properties and thus demonstrating the need of Fe into the tetrahedra.

Despite the expected weak difference as a magnetic link between F^−^ *vs*. OH^−^,^[^ ^22^ ^]^ DFT + *U* (*U* = 6 eV) calculations show strong distinctions between the three identified intra‐layer *J*_O‐X_ (*J*_1_), *J*_O‐O_ (*J*_2_), *J*_X‐X_ (*J*_3_) exchange interactions (see Figure 1a) for diamagnetic tetrahedral sites. These are in favor of “frustrated AFM” for *X*═F^−^ against FM for *X*═OH^−^, see Supporting Information. This FM character is reinforced when 25% of Fe^3+^ is incorporated in the tetrahedral layer due to significant *J*_Tetra‐Octa_ (*J*_T‐O_) AFM exchange, forcing the octahedral moments to align antiparallel with the tetrahedra but parallel within themselves.

Concerning the inter‐layer magnetic exchanges (*J_inter_*), the presence of tetrahedral Fe^3+^ locally reduces the interlayer distance for magnetic interactions from ≈10 Å to ≈5.8 Å. However, *J*_inter_ is calculated as one or two orders of magnitude smaller than the intralayer ones, for none, one or two Fe tetrahedral layers mediating the exchange between the *S* = 2 triangular lattices.

Thus, once the frustration is broken by the *J*_T‐O_ exchanges into ferrimagnetic TOT spin slabs, the coupling between successive layers may occur through magnetic dipole–dipole interactions, as observed in for instance Cs_2_AgF_4_ and several other layered magnetic systems.^[^ ^23^ ^]^

## 3 Conclusion

Discovering the thermal evolution from 2D to 3D long‐range magnetic order in KFe_3_^2+^[FeGe_3_]O_10_(OH)_2_ synthetic mica marks an important milestone in developing magnetic applications in this vast mineral family, as it is demonstrated by the main role of the iron occupancy in the tetrahedral layer breaking the frustration in the triangular network which is crucial in developing 3D magnetic ordering. Thus, 2D FM octahedral layers weakly coupled order at high temperature and as cooling down they induce the AFM ordering in the tetrahedral Fe^3+^ layer which at the same time forces the 3D ordering observed via dipole‐dipole interactions.

## Conflict of Interest

The authors declare no conflict of interest.

## Supporting information



Supporting Information

## Data Availability

The data that support the findings of this study are available from the corresponding author upon reasonable request.
