# Supplementary material for: 2D to 3D Magnetism in Synthetic Micas
Source: Adv Sci (Weinh). 2024 Sep 20;11(42):2408266. doi: 10.1002/advs.202408266 (PMC11558087; doi:10.1002/advs.202408266)
Supplement: Supplementary file 1 — Supporting Information [file ADVS-11-2408266-s001.docx]

Supplementary Information for

2D to 3D magnetism in synthetic micas.

Jose Luis Rosas-Huerta,*^[a]^ Jonas Wolber,^[a,b]^, Claire Minaud,^[a]^ Oscar Fabelo,^[b]^ Clemens Ritter,^[b]^ Olivier Mentré, ^[a]^ and Ángel M. Arévalo-López*^[a]^

[a] Dr. J.L. Rosas-Huerta, J. Wolber, C. Minaud, Dr. O. Mentré, Dr. A.M. Arévalo-López
Unité de Catalyse et Chimie du Solide (UCCS) – UMR CNRS 8181

Université de Lille – Centrale Lille, Université Artois, ENSCL, Lille, F-59000, France.
E-mail: [joseluis_rosas@comunidad.unam.mx](mailto:joseluis_rosas@comunidad.unam.mx), [angel.arevalo-lopez@univ-lille.fr](mailto:angel.arevalo-lopez@univ-lille.fr)

[b] Dr. O. Fabelo, Dr. C. Ritter
Institut Laue-Langevin,

BP 156, 38042 Grenoble Cedex, France.

**Synthesis details**

The KFe_3_[FeGe_3_]O_10_(OH)_2_ compounds were prepared under pressure by the hydrothermal synthesis method. Stoichiometric amounts of GeO_2_, Fe_2_O_3_ and K_2_CO_3_ were mixed and introduced together with 0.8 ml of a 0.5 M KOH solution into a 0.5 mm diameter silver tube of 10 cm of length which was then welded. The temperature was increased slowly until the system reached 773 K and a pressure of 1.7 kbar, 60 hours later it was turned off and allowed to cool down. The product was composed of conglomerates of translucent plates and red powder that were identified as the micas and Fe_2_O_3_, respectively. The mica crystals were washed with distilled water and then dried.

For the fluoride mica compounds KFe_3_[FeGe_3_]O_10_F_2_ and KFe_3_[GaGe_3_]O_10_F_2_, stoichiometric amounts of KF, FeO, Fe_2_O_3_, Ga_2_O_3_, FeF_2_ and GeO_2_ were weighed under Ar atmosphere. The powders were mixed, pressed and introduced in a 0.5 mm diameter gold tube of 4 cm of length. These were introduced in quartz tubes and sealed under vacuum. The compounds were heated at 973 K for 10 h and slowly cool down to room temperature.

**Diffraction experiments details.**

Single crystal X-ray diffraction data were done in a X8-Bruker APEX DUO diffractometer (λ = Mo_kα_ and Cu_kα_). APEX2 Suite software was implemented to extract and correct the intensities [^[[1]](#endnote-1)^]. Multi-scan absorption corrections were applied using the SADABS program [^[[2]](#endnote-2)^]. The structure solution was performed in JANA2000 [^[[3]](#endnote-3)^]. Structural data are shown in Table S1. Synchrotron x-ray radiation (λ = 0.824899 Å) experiments were made in the BL04 MSPD beamline of ALBA synchrotron in Spain in the 2 ° – 60 ° range with a 0.004 ° step in 2θ. Neutron powder diffraction were made at the Institut Laue-Langevin (ILL) in the D20 beamline with λ = 1.54 Å (90° take-off angle) for high resolution, while λ = 2.42 Å were used for high flux. Rietveld refinements were done using the Fullprof suite [^[[4]](#endnote-4)^]. The possible magnetic structures and space groups were identified by ISODISTORT [^[[5]](#endnote-5)^ ^[[6]](#endnote-6)^].


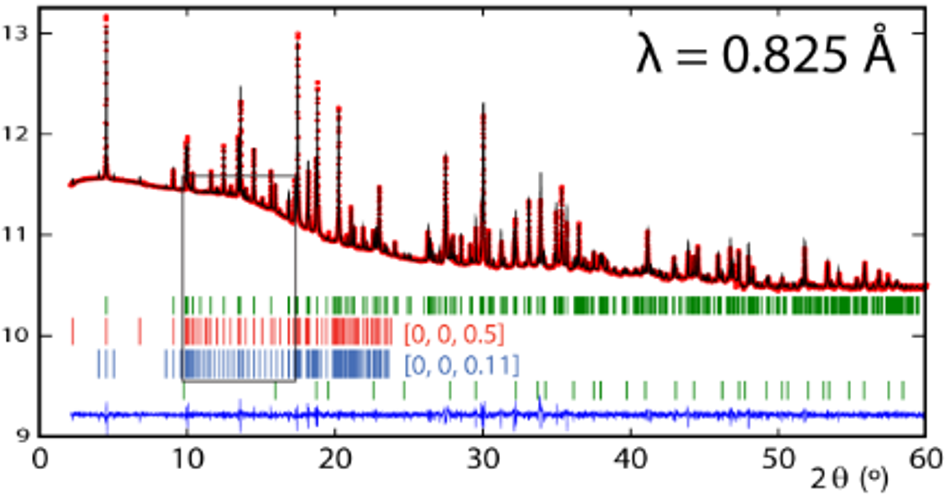


**Figure S1.** Full Rietveld refinement made to the SXRD for the KFe_3_[FeGe_3_]O_10_(OH)_2_ mica compound at 300 K (λ = 0.825 Ả) with red and blue marks indicating 2c and 9c supercells. The rectangle indicates the enlarged region showed in Figure 1 in the main text.

**Warren function fitting.**


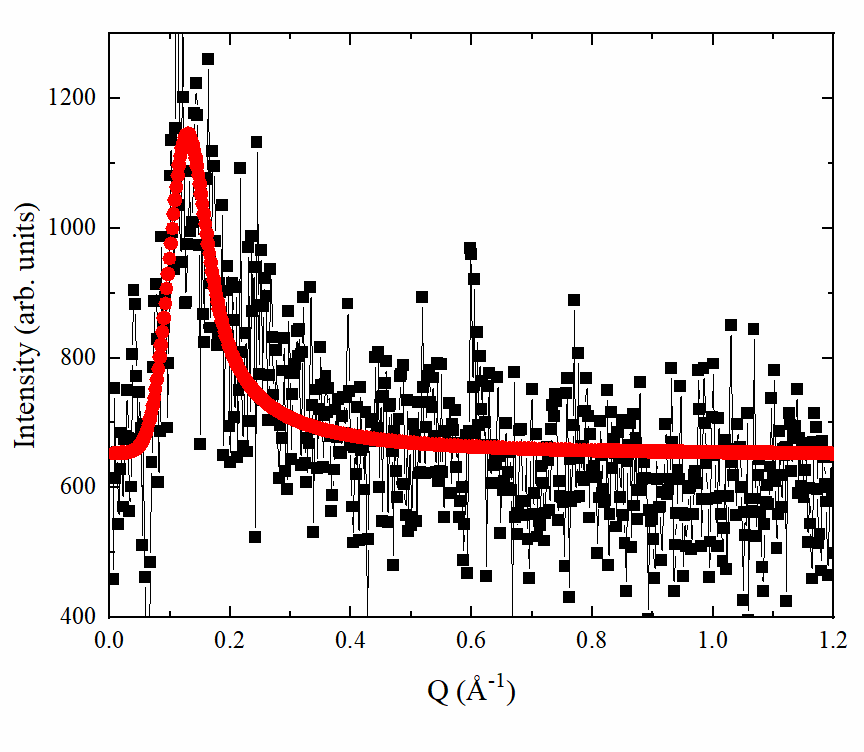


**Figure S2.** 60 K minus 80 K NPD difference pattern, square black dots and solid red line shows the experimental data and the Warren function fitting, respectively.

The Warren function was modeled by the equation:

$P\left( Q \right)=Km\frac{F_{hk}^{2}\left[ 1-2\left( \frac{\lambda Q}{4\pi} \right)^{2}+2\left( \frac{\lambda Q}{4\pi} \right)^{4} \right]}{\left( \frac{\lambda Q}{4\pi} \right)^{\frac{3}{2}}}\times\left( \frac{\xi}{\lambda\sqrt{\pi}} \right)^{\frac{1}{2}}F(a)\left[ f(Q) \right]^{2}$,

where $P\left( Q \right), K, m, F_{hk}, \lambda, Q, \xi$ and $f\left( Q \right)$ are the static structure factor, scaling constant, multiplicity, two-dimensional structure factor for the spin array, wavelength, scattering wave, spin-spin correlation length and magnetic form factor, respectively with with $a=\frac{\xi\sqrt{\pi}}{2\pi}\left( Q-Q_{0} \right)$ and $F\left( a \right)=\int_{0}^{10} exp\left[ -{(x^{2}-a)}^{2} \right]dx$ where $Q_{0},$ is the center of the peak and x is delimited to 10 to make the integral converge.


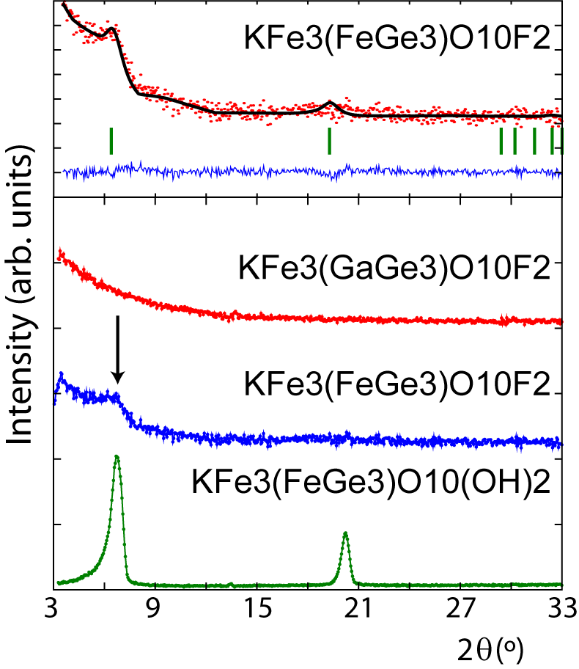


**Figure S3**. NPD difference patterns (λ = 2.42 Å) between high and low temperature for the fluoride micas KFe_3_[FeGe_3_]O_10_F_2_ (1.6 K - 24 K) and KFe_3_[GaGe_3_]O_10_F_2_ (1.6 K - 50 K) and hydroxy mica KFe_3_[FeGe_3_]O_10_(OH)_2_ (10 K - 80K). At the top it shows the Rietveld refinement for the difference in KFe_3_[FeGe_3_]O_10_F_2_.


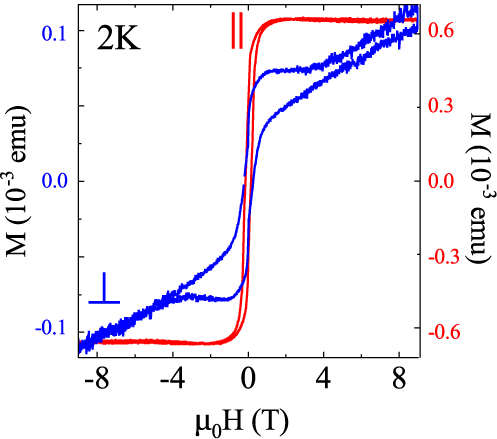


**Figure S4**. Magnetization as function of the applied magnetic field in the parallel (‖, red color) and perpendicular (⊥, blue color) orientations at 2 K for mica KFe_3_[FeGe_3_]O_10_(OH)_2_ single crystal. The units were left in emu since the mass of the crystal was unknown.


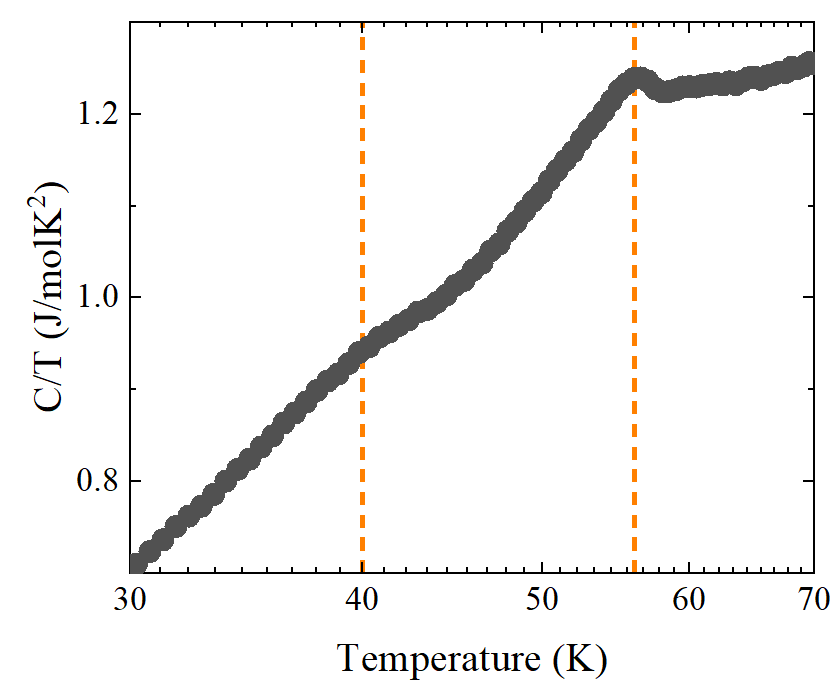


**Figure S5**. Total molar heat capacity divided by temperature (C/T) as temperature dependence. Dotted orange lines show the magnetic *T_incom_* = 56 K and *T_C_* = 40 K transitions.





**Figure S6**. Rietveld refinement to the high resolution NPD patterns (λ = 1.54) of the KFe_3_[FeGe_3_]O_10_(OH)_2_, KFe_3_[FeGe_3_]O_10_F_2_ and high flux (λ = 2.42 Å) for KFe_3_[GaGe_3_]O_10_F_2_ over their magnetic transition.


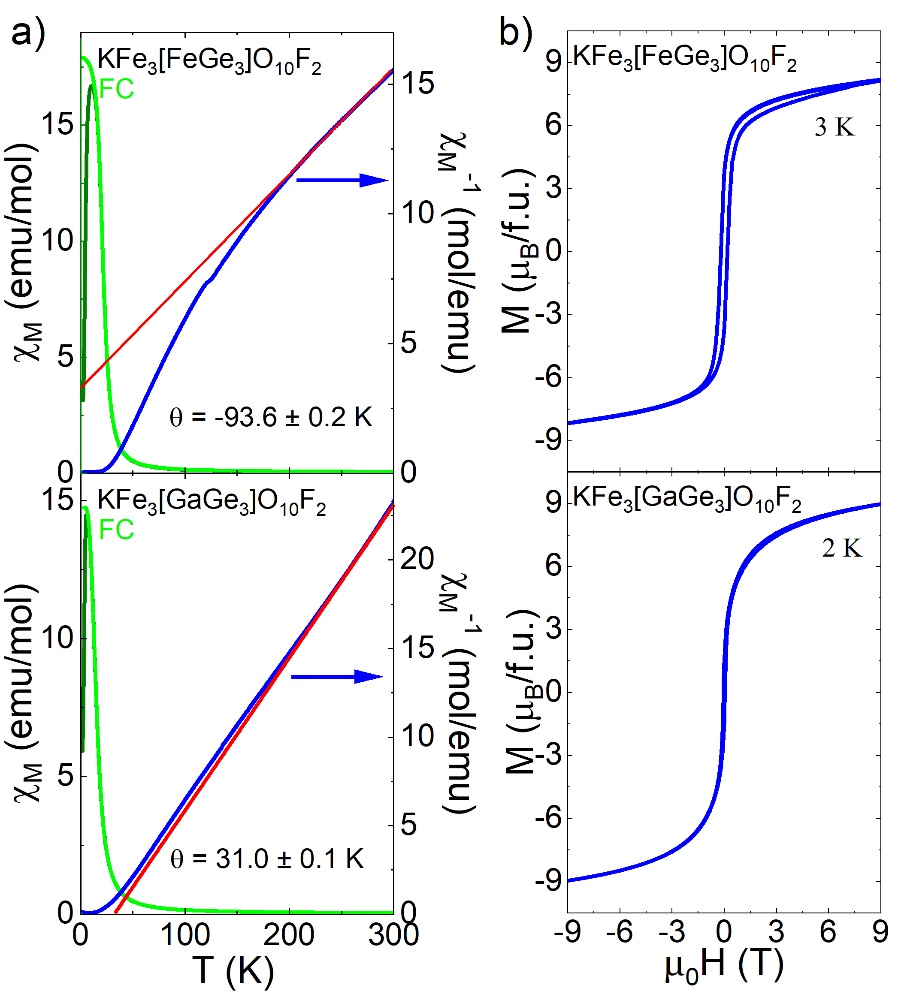


**Figure S7**. a) FC/ZFC magnetic susceptibilities and inverse ZFC data with Curie-Weiss fit to 150-300 K points, b) M vs H at low temperature in KFe_3_[FeGe_3_]O_10_F_2_ and KFe_3_[GaGe_3_]O_10_F_2_ powder samples.

Table S1. Monocrystal structure data from K_0.968_Fe_3.968_Ge_2.905_O_10_(OH)_2_ at 293 K.

| **Crystal data (300 K)** | |
| --- | --- |
| Formula | KFe_4_Ge_3_O_10_(OH)_2_ |
| Molar weight (g/mol) | 664.3 |
| Symmetry | Monoclinic |
| Space group | *C*2*/m*, no. 12 |
| Z | 2 |
| Lattice parameters (Å)  (°) | *a* = 5.5203 (3)  *b* = 9.5626 (6)  *c* = 10.5975 (6)  *β* = 100.049 (4) |
| **Data collection** | |
| Equipment | X8-Bruker APEX DUO |
| λ (Mo_Kα_ (graphite monochromator); Å) | 0.71073 |
| density calc. (g/cm^3^) | 4.005 |
| Color | transparent yellow |
| Scan mode | 𝜔, φ |
| θ (min-max) (°) | 1.95 – 25.24 |
| μ (mm^-1^; for λ Kα = 0.7107 Å) | 13.323 |
|  |  |
| *R*(int) (%) | 5.87 |
| Recording reciprocal space | -9 ≤ *h* ≤ 8, -17 ≤ *k* ≤ 16, -19 ≤ *l* ≤ 19 |
| Number of measured reflections | 18244 |
| Number of independent reflections (*I* > 3σ(*I*)), total | 1111/1277 |
| **Refinement** | |
| Number of refined parameters | 48 |
| Refinement method, program | Least squares on F |
| Twin fractions | 52.9/22.9/24.2 |
| *R*1(*F*)[ *I* > 2*σ*(*I*)]/*R*1(*F*^2^) [all data, %] | 4.88/5.41 |
| *wR*2(F^2^) [ *I* > 2*σ*(*I*)]/*wR*2(*F*^2^) [all data, %]  *w* = 1/( *σ*^2^(F_o_^2^) + (0.0682P)^2^) | 6.47/6.57 |
| GOF | 2.23 |
| Max/Min residual electronic density (e^-^/ Å^3^) | 1.40/-0.69 |

**Table S2**. Crystallographic data obtained from the Rietveld fitting made to the synchrotron x-rays diffraction measurements (λ = 0.824899 Å) for the KFe_4_Ge_3_O_10_(OH)_2_ at 293 K. BVS ($s_{ij}$) was obtained considering the relation $s_{ij}=exp[(r_{0}-r_{ij})/b]$, where $r_{ij}$ are the bond lengths, $b$ = 0.37 and $r_{0}$ is an empirical parameter.

| Atom | Wyckoff  position | x | y | z | U_ani_ | BVS |
| --- | --- | --- | --- | --- | --- | --- |
| Ge1/Fe3* | 8d | 0.4253(2) | 0.1667(5) | 0.2736(1) | 0.010 (3) | 3.66 |
| Fe1 | 4g | 0.0 | 0.1672(1) | 0.0 | 0.011(1) | 1.92 |
| Fe2 | 2b | 0.5 | 0.0 | 0.0 | 0.012(1) | 1.93 |
| K | 2c | 1.0 | 0.0 | 0.5 | 0.026(1) | 0.83 |
| O1 | 8j | 0.366(2) | 0.1669(3) | 0.1051(3) | 0.011(1) |  |
| OH2 | 4i | 0.862(12) | 0.0 | 0.010(1) | 0.018(2) |  |
| O3 | 4i | 0.5341(9) | 0.0 | 0.3344(5) | 0.020(2) |  |
| O4 | 8j | 0.6515(8) | 0.2952(4) | 0.3349(4) | 0.023(2) |  |
| H1 | 4d | 0.9652 | 0.0 | 0.1672 | 0.0216 |  |
|  | |  |  |  |  |  |
| Bond distance (Å) | | | Bond angle (°) | | |  |
| Ge1/Fe3-O1 (a) | | 1.759(4) | Fe1–O1–Fe2 ×2 | | 93.4(2) |  |
| Ge1/Fe3-O3 (b) | | 1.784(2) | Fe1–O1–Fe1 ×4 | | 96.3(2) |  |
| Ge1/Fe3-O4 (b) | | 1.792(4) | Fe1–O1–Fe2 ×2 | | 95.7(4) |  |
| Ge1/Fe3-O4 (b) | | 1.784(5) | Fe2–OH2–Fe1 ×4 | | 98.2(3) |  |
| Fe1-O1 ×2 | | 2.130(10) | Fe1–OH2–Fe1 ×2 | | 97.3(4) |  |
| Fe1-O1 ×2 | | 2.143(6) |  |  |  |  |
| Fe1-OH2 ×2 (cis) | | 2.130(6) |  |  |  |  |
| Fe2-O1 ×4 | | 2.150(6) |  |  |  |  |
| Fe2-OH2 ×2 (trans) | | 2.091(10) |  |  |  |  |
| K-O3 ×2 | | 2.849(5) |  |  |  |  |
| K-O4 ×4 | | 2.847(4) |  |  |  |  |

*Ge1/Fe3 are in 0.75/0.25 occupancy

**Table S3**. Lattice parameters obtained from the Rietveld fitting made to the NPD high resolution (λ = 1.54 Å) for the micas KFe_3_[FeGe_3_]O_10_(OH)_2_, KFe_3_[FeGe_3_]O_10_F_2_ and KFe_3_[GaGe_3_]O_10_F_2_.

| Mica composition | Temperature (K) | Lattice parameters | | | | |
| --- | --- | --- | --- | --- | --- | --- |
|  |  | a (Å) | b (Å) | c (Å) | β (°) | Volume (Å^3^) |
| KFe_3_[FeGe_3_]O_10_(OH)_2_ | 150 | 5.4858(5) | 9.5192(9) | 10.5211(7) | 99.741(7) | 541.50(8) |
| KFe_3_[FeGe_3_]O_10_F_2_ | 100 | 5.4535(9) | 9.4535(9) | 10.4440(8) | 100.220(9) | 530.40(9) |
| KFe_3_[GaGe_3_]O_10_F_2_ | 50 | 5.4806(10) | 9.4799(14) | 10.4043(14) | 100.737(11) | 531.10(14) |

**DFT calculations details.**

Calculations were made in the framework of the Density Functional Theory (DFT) by the Vienna Ab initio Simulation Package (VASP). [^[[7]](#endnote-7)^] Generalized Gradient Approximation was implemented with the Hubbard correction (GGA + U, U = 6 eV) for correlated 3*d* orbitals in iron atoms with the PBE exchange correlation functional [^[[8]](#endnote-8)^] and the Duradev method. [^[[9]](#endnote-9)^] One formula with the triclinic primitive cell with lattice parameters *a* = 5.50053 Å, b = 5.49955 Å, c = 10.17121 Å, α = 95.4174°, β = 95.1011° and γ = 120.1512 was used for the geometric optimization without symmetry restrictions to make spin polarized calculations with the following convergence criteria: 400 eV by cut-off energy for plane wave basis set, minimum energy difference of 1×10^-8^ eV in the self-consistent field method and a *k*-point grid of 8×8×4 in the first Brillouin zone.

To study the super-exchange interactions (*J*) between iron atoms in the trioctahedral mica compounds, supercells were created as it is shown in the Figure S7 a) and b). The interaction effect between triangular layers composed by Fe (*J*_inter_) was studied by the *a*×*b*×2*c* supercell with and without iron in tetrahedra layer (KFe_3_[GeGe_3_]O_10_(OH)_2_ and KFe_3_[FeGe_3_]O_10_(OH)_2_) and a 2*a*×*b*×2*c* supercell for iron in the two tetrahedra layers (KFe_3_[FeGe_3_]O_10_(OH)_2_). The exchange interaction between the iron in tetrahedra and octahedra (*J*_T-O_) was studied to replace one iron in two tetrahedra layers. It was calculated with iron located at the 1, 2, 3 and 4 tetrahedra sites in the 2*a*×*b*×2*c* supercell, see Figure S7a. For the the iron intralayer interactions (*J_intra_*), three different AFM configurations were calculated in a 2*a*×*b*×*c* supercell, see Figure S7b. For these, four iron atoms were assigned magnetic moments of 7 µ_B_, while two were spin directed in opposite direction with -7 µ_B_ for the AFM1 (B and E octahedra sites), AFM2 (A and D octahedra sites) and AFM3 (A and E octahedra sites).These magnetic configurations help to obtain three different *J*_intra_ label as *J*_1_, *J*_2_ and *J*_3_ which are identified for the Fe interactions between oxygen - fluor, oxygen - oxygen and fluor - fluor, respectively.


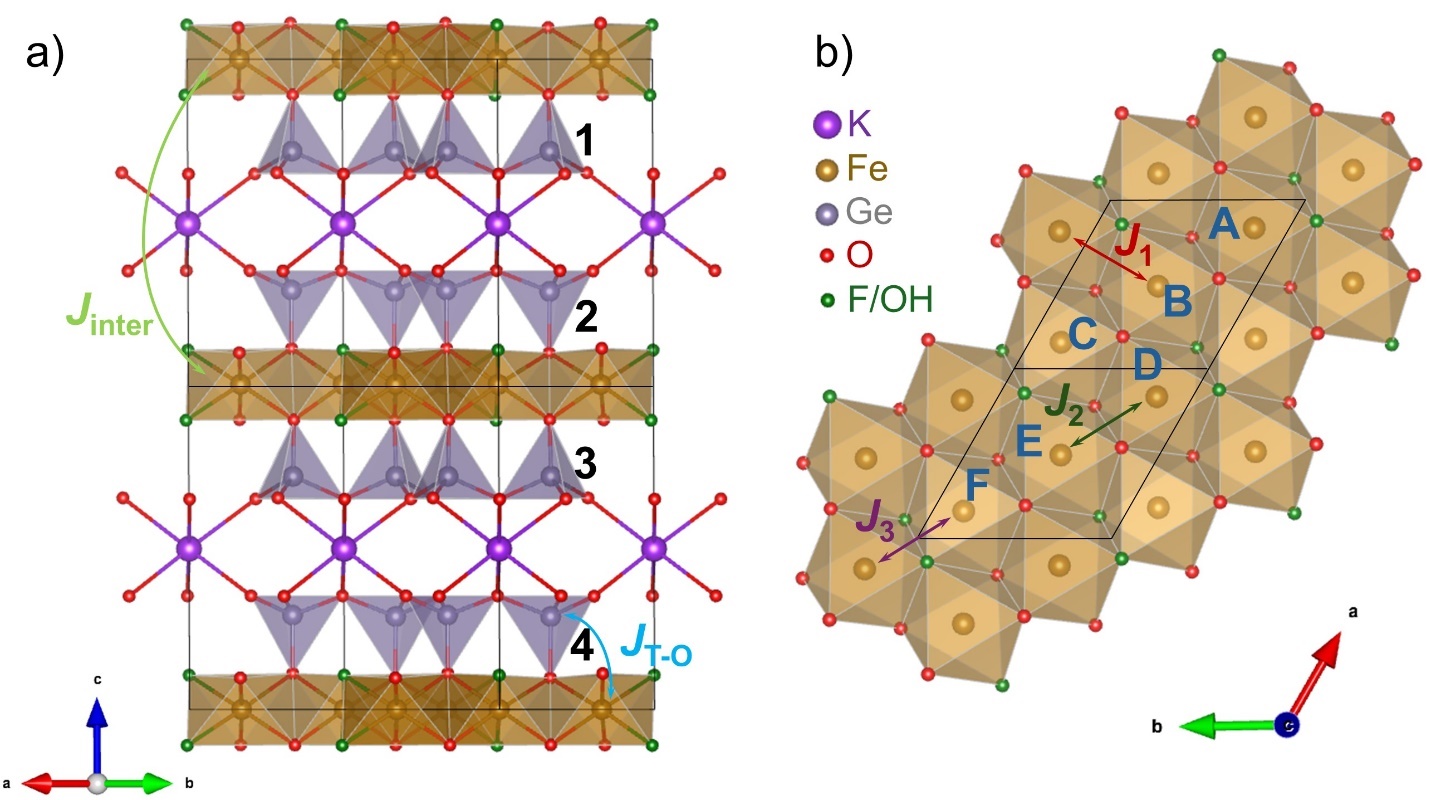


**Figure S8**. a) 2*a*×*b*×2*c* and b) 2*a*×*b*×*c* supercell models to calculate *J*s values. Description is in the text.

The *J*s can be extracted from the relation of the spin Hamiltonian for the ordered spin states $\hat{H}=-\sum_{i<j} J_{ij}\hat{S_{i}}\cdot\hat{S_{j}}$ with $J_{ij}$ as the *J*_1_, *J*_2_ and *J*_3_ for the *i* and *j* spin site. Then the $J_{\mathrm{inter}}$ in mica without iron in tetrahedra sites (*a*×*b*×2*c*) can be obtained by the relation $E_{\mathrm{FM}}-E_{\mathrm{AFM}}=12J_{\mathrm{inter}}\cdot4^{2}/4$, while for the iron in the two tetrahedra layers (2*a*×*b*×2*c* or 2*a*×*b*×*c*) the AFM arrangement: $E_{\mathrm{FM}}-E_{\mathrm{AFM}}=24J_{\mathrm{inter}}\cdot{4.25}^{2}/4$. It is worth mentioning that the $E_{\mathrm{AFM}}$ in the *a*×*b*×2*c* the triangular layers are in opposite direction, while the 2*a*×*b*×2*c* or 2*a*×*b*×*c* is related to the arrangement where triangular layers are in FM configuration and the iron in tetrahedra is in opposite direction as it was found in experimental results. For the 2*a*×*b*×*c* supercell (KFe_3_[GeGe_3_]O_10_(OH)_2_) the *J*s interactions were determined by solving the system of equations:

$$E_{\mathrm{FM}}-E_{AFM1}={(16J}_{1}+{8J}_{2})\cdot4/4^{2}$$

$$E_{\mathrm{FM}}-E_{AFM2}={(8J}_{1}+{12J}_{2}{+4J}_{3})\cdot4/4^{2}$$

$$E_{\mathrm{FM}}-E_{AFM3}={(8J}_{1}+{10J}_{2}{+2J}_{3})\cdot4/4^{2}$$

*J*s values and the Curie-Weiss temperature ($T_{CW}=\sum J_{i}S(S+1)/3k_{B}$) are listed in Table S4

**Table S4**. Js and $T_{CW}$ for mica compounds with and without iron in tetrahedra layers as described in the text. $T_{CW+inter}$ considers the $J_{inter}$.

|  |  | KFe_3_[GaGe_3_]O_10_F_2_ | KFe_3_[GaGe_3_]O_10_(OH)_2_ |
| --- | --- | --- | --- |
|  |  | supercell 2*a*×*b*×2*c* | supercell 2*a*×*b*×2*c* |
| relative energies | AFM1 | -0.02379 | 0.00710 |
| (eV/unit cell) | AFM2 | 0.00707 | 0.00573 |
|  | AFM3 | -0.05119 | -0.02973 |
|  | AFM 2c* | -0.00259 | 0.00150 |
|  | AFM 2a** | - | 0.13481 |
| Spin exchange | *J*_1_ | 62.1 | 52.4 |
| parameters (K) | *J*_2_ | -141.5 | -99.7 |
|  | *J*_3_ | 310.6 | 202.6 |
|  | *J*_inter_ | -1.3 | 0.7 |
|  | *J_T-O_* | - | 57.7 |
| T_CW_ (K) |  | 9.3 | -18.0 |
| T_CW+inter_ (K) |  | 14.3 | -20.8 |
|  |  | Fe in 2Td | Fe in 2Td |
|  |  | supercell 2*a*×*b*×2*c* | supercell 2*a*×*b*×*c* |
| relative energies (eV/unit cell) | AFM  AFM T_d_ | 0.00054  0.14329 | -  0.13481 |
| Spin exchange parameters (K) | *J*_inter_  *J*_T-O_ | 0.2  61.4 | -  57.7 |

*Energy extracted from a supercell *a*×*b*×2*c*. **Energy extracted from a supercell 2*a*×*b*×*c*.

1. Bruker (**2010**). APEX2 Suite. Bruker AXS Inc., Madison, Wisconsin, USA. [↑](#endnote-ref-1)
2. SADABS: Area-Detector Absorption Correction; Siemens Industrial. Automation, Inc.: Madison, WI, **1996**. [↑](#endnote-ref-2)
3. V. Petricek, M. Dusek, L. Palatinus, JANA2006, Institute of Physics, Academy of Sciences, Praha, Czech Republic, **2006**. [↑](#endnote-ref-3)
4. 27 J. Rodríguez-Carvajal, *Physica B* **1993**, *192*, no. *(1–2)*, 55. [↑](#endnote-ref-4)
5. H. T. Stokes, D. M. Hatch, and B. J. Campbell, “ISODISTORT, ISOTROPY Software Suite,” ISODISTORT, ISOTROPY, Software Suite, iso.byu.edu. [↑](#endnote-ref-5)
6. B. J. Campbell, H. T. Stokes, D. E. Tanner, and D. M. Hatch, *J. Appl. Crystallogr.* **2006**, *39* no. *(4*. *)*, 607. [↑](#endnote-ref-6)
7. G. Kresse, and J. Hafner, *Phys. Rev. B* **1993**, *47*, 558 [↑](#endnote-ref-7)
8. J. P. Perdew, K. Burke, M. Ernzerhof, *Phys. Rev. Lett.* **1996**, *77*, 3865. [↑](#endnote-ref-8)
9. S. L. Dudarev, G. A. Botton, S. Y. Savrasov, C. J. Humphreys, A. P. Sutton, *Phys. Rev. B* **1998**, B *57*, 1505. [↑](#endnote-ref-9)
